# APRANK: Computational Prioritization of Antigenic Proteins and Peptides From Complete Pathogen Proteomes

**DOI:** 10.3389/fimmu.2021.702552

**Published:** 2021-07-15

**Authors:** Alejandro D. Ricci, Mauricio Brunner, Diego Ramoa, Santiago J. Carmona, Morten Nielsen, Fernán Agüero

**Affiliations:** ^1^ Instituto de Investigaciones Biotecnológicas “Rodolfo Ugalde” (IIB), Universidad de San Martín (UNSAM) – Consejo Nacional de Investigaciones Científicas y Técnicas (CONICET), Buenos Aires, Argentina; ^2^ Department of Health Technology, The Technical University of Denmark, Lyngby, Denmark

**Keywords:** antigens, linear epitopes, antigenicity, prediction, human pathogens

## Abstract

Availability of highly parallelized immunoassays has renewed interest in the discovery of serology biomarkers for infectious diseases. Protein and peptide microarrays now provide a rapid, high-throughput platform for immunological testing and validation of potential antigens and B-cell epitopes. However, there is still a need for tools to prioritize and select relevant probes when designing these arrays. In this work we describe a computational method called APRANK (Antigenic Protein and Peptide Ranker) which integrates multiple molecular features to prioritize potentially antigenic proteins and peptides in a given pathogen proteome. These features include subcellular localization, presence of repetitive motifs, natively disordered regions, secondary structure, transmembrane spans and predicted interaction with the immune system. We trained and tested this method with a number of bacteria and protozoa causing human diseases: *Borrelia burgdorferi* (Lyme disease), *Brucella melitensis* (Brucellosis), *Coxiella burnetii* (Q fever), *Escherichia coli* (Gastroenteritis), *Francisella tularensis* (Tularemia), *Leishmania braziliensis* (Leishmaniasis), *Leptospira interrogans* (Leptospirosis), *Mycobacterium leprae* (Leprae), *Mycobacterium tuberculosis* (Tuberculosis), *Plasmodium falciparum* (Malaria), *Porphyromonas gingivalis* (Periodontal disease), *Staphylococcus aureus* (Bacteremia), *Streptococcus pyogenes* (Group A Streptococcal infections), *Toxoplasma gondii* (Toxoplasmosis) and *Trypanosoma cruzi* (Chagas Disease). We have evaluated this integrative method using non-parametric ROC-curves and made an unbiased validation using *Onchocerca volvulus* as an independent data set. We found that APRANK is successful in predicting antigenicity for all pathogen species tested, facilitating the production of antigen-enriched protein subsets. We make APRANK available to facilitate the identification of novel diagnostic antigens in infectious diseases.

## Introduction

Infectious diseases are one of the first causes of death worldwide, disproportionately affecting poor and young people in developing countries. Several epidemiological and medical strategies exist to deal with these diseases, most of which rely on robust and accurate diagnostic tests. These tests are used to demonstrate infection (presence of the pathogen), to follow up treatments and to monitor the evolution or cure of the disease or the success of field interventions (1).

One of the preferred methods to diagnose infections relies on the detection of pathogen-specific antibodies in the fluids of infected patients (most often serum obtained from blood) (2, 3). For this reason, there is a big interest in developing reliable methods able to improve the fast and sensitive identification of potential specific antigens.

With the advent of peptide microarray platforms it is now possible to perform high-throughput serological screening of short peptides, which allows for faster discovery of linear antigenic determinants with good potential for diagnostic applications (4). Taking advantage of complete genome sequences from pathogens, it is theoretically possible to scan every encoded protein with short peptides against sera from infected hosts. However, while this is straightforwardly achieved for viral pathogens and small bacteria, it gets more difficult when dealing with larger bacteria or eukaryotic parasites, since they can reach thousands of proteins with millions of peptides, exceeding the average capacity of standard protein or peptide microarrays (5). Besides, it is now becoming common to fit in the arrays additional sequence variants obtained from the pathogen population (from diverse strains and clinical isolates). One example are serological strain typing strategies (6), which would stress the capacity of these platforms.

Ultrahigh-density peptide microarrays had been used successfully to map linear epitopes, having an upper theoretical limit of ~ 2-3 million unique peptides per array (7). While these ultrahigh-density peptide microarrays do enable a lot of possibilities, they do not yet have the capacity to analyze whole proteomes of larger pathogens without some preprocessing. It is also worth noting that they are not widely available as lower density arrays and they require substantial processing and downstream work to deal with large proteomes (8–10).

There are several ways to deal with the problem of not having enough space when accommodating large proteomes in a peptide array, each with their own advantages and disadvantages. The most common are: decreasing the overlap between peptides, dividing the proteome among different microarray slides, and using computational methods to prioritize antigens. In this paper we will focus on the latter. We and others have previously shown that a number of protein features can be used to validate and prioritize candidate antigens and epitopes for human pathogens (8, 11–13). Similar approaches have also been developed into a number of reverse vaccinology programs for bacteria [reviewed recently in Dalsass et al. (14)].

In a previous work, we developed a method that integrates information from a number of calculated molecular and structural features to compute an antigenicity score for proteins and peptides in *Trypanosoma cruzi* (8, 11). In this paper, we use machine learning techniques to extend and generalize this concept so that it can be applied to other pathogens. We call this method APRANK (Antigenic Protein and Peptide Ranker) and show how it can be used as a strategy to predict and prioritize diagnostic antigens for several human pathogens.

## Materials and Methods

All methods are described in detail herein, but are also documented as R and Perl code in APRANK’s source code, available at GitHub (see *Availability*).

### Bioinformatic Analysis

FASTA files containing proteins of the species used to train APRANK (see **Table 1**) were downloaded from publicly available database resources (from complete proteomes) and can be found in the GitHub repository (see *Availability*). To comply with requirements of downstream predictors, unusual amino acid characters were replaced by the character ‘X’ and a few proteins with more than 9,999 amino acids were truncated to that size. To obtain information at peptide level, proteins were split into peptides of 15 residues with an overlap of 14 residues between them (meaning an offset of 1 residue between peptides).

**Table 1:** List of pathogen species used in this paper.

Pathogen Species	Disease	Group	Taxonomy (Phylum)
*Borrelia burgdorferi*	Lyme disease	Gram Negative Bacteria	Spirochaetia
*Brucella melitensis*	Brucellosis		Alpha-proteobacteria
*Coxiella burnetii*	Q fever		Gamma-proteobacteria
*Escherichia coli*	Gastroenteritis		Gamma-proteobacteria
*Francisella tularensis*	Tularemia		Gamma-proteobacteria
*Leptospira interrogans*	Leptospirosis		Spirochaetia
*Porphyromonas gingivalis*	Periodontal disease		Bacteroidetes
*Mycobacterium leprae*	Leprosy	Gram Positive Bacteria	Actinobacteria
*Mycobacterium tuberculosis*	Tuberculosis		Actinobacteria
*Staphylococcus aureus*	Bacteremia		Firmicutes
*Streptococcus pyogenes*	GAS infections		Firmicutes
*Leishmania braziliensis*	Leishmaniasis	Eukaryotic Protozoa	Euglenozoa
*Plasmodium falciparum*	Malaria		Apicomplexa
*Toxoplasma gondii*	Toxoplasmosis		Apicomplexa
*Trypanosoma cruzi*	Chagas Disease		Euglenozoa

The validated FASTA files were analyzed with BepiPred (15), EMBOSS pepstats, Iupred (16), NetMHCIIpan (17), NetOglyc (18), NetSurfp (19), Paircoil2 (20), PredGPI (21), SignalP (22), TMHMM (23), Xstream (24) and two custom perl scripts that analyzed similarity of short peptides against the human genome (NCBI BioProject PRJNA178030). The reasoning of choosing each predictor, what they predict and which version was used can be found in **Table 2**. The full console call for each predictor can be seen in **Supplementary Table S2**. NetMHCIIpan was run multiple times for different human alleles (DRB1*0101, DRB3*0101, DRB4*0101 and DRB5*0101). The only predictor that needed an extra preprocessing step was PredGPI, which required removing sequences shorter than 41 amino acids and those with an ‘X’ in their sequence. For all purposes, these filtered sequences were assumed to not have a GPI anchor signal. The versions of Linux, R, Perl, packages and modules used to create the computational method are listed in **Supplementary Table S1**.

**Table 2:** Predictors used to analyze different features of proteins and peptides.

Focus	Feature	Predictor	Basis
Stimulation of an immune response	B-cell epitopes	BepiPred 1.0	Antigenicity by HMM
	Binding to MHC Class II molecules	NetMHCIIpan 2.0	ANN trained with peptide and MHC Class II sequence information
Peculiarities in the protein sequence	Glycosylation sites	NetOglyc 3.1d	ANN trained with mucin type GalNAc O-glycosylation sites in mammalian proteins
	GPI-anchored proteins	PredGPI 1.4.3	Discrimination of the anchoring signal by SVM and prediction of the most probable omega-site by HMM
	Signal peptide cleavage sites	SignalP 4.0	Prediction of cleavage sites and a signal peptide/non-signal peptide prediction based on a combination of several ANN
	Tandem repeats	Xstream 1.71	SE algorithm to explicitly locate exact and degenerate tandem repeats TRs of all periods in protein sequences
Three dimensional structure	Disorder	Iupred 1.0	Amino acids favorable interactions potential
	Parallel coiled coil fold	Paircoil2	Uses pairwise residue probabilities with the Pair coil algorithm and an updated coiled coil database
	Secondary Structure	NetSurfp 1.0	ANN trained with sequence profiles and predicted secondary structure
	Surface access	NetSurfp 1.0	ANN trained to predict the relative surface exposure of the individual amino acid residues
	Transmembrane helices in proteins	TMHMM 2.0c	Membrane protein topology prediction method based on a HMM
Molecular properties	Isoelectric point	Pepstats (EMBOSS 6.6.0.0)	Amino acids pK values
	Molecular Weight	Pepstats (EMBOSS 6.6.0.0)	Amino acids weights
Similarities within itself and with the host	Sequence similarity (pathogen/host)	CrossReactivity	Shared kmers between pathogen and host proteins
	Sequence similarity (pathogen proteins)	SelfSimilarity	Shared kmers between pathogen proteins

CrossReactivity and SelfSimilarity are custom Perl scripts. ANN, Artificial Neural Network; HMM, Hidden Markov Model; SE, Seed Extension; SVM, Support Vector Machine.

### Compiling a Dataset of Curated Antigens

To obtain antigenic proteins and peptides, we extracted information from the immune epitope database (IEDB), as well as information from several papers, most of which relied on data from protein or peptide microarrays combined with sera of infected patients to find new antigens (11, 25–40).

Because different protein identifiers are used across papers, we used either the Uniprot ID mapping tool, the blastp suite of BLAST or a manual mapping to find the corresponding ID or IDs that a given antigen had in our proteomes. The exhaustive list of all antigenic proteins and peptides used, their source and the mapping method used can be found in the Supplementary Data accompanying this article. A version of these data with only the antigenicity information can be found in the GitHub repository (see *Availability*).

For the antigenic peptides, though, mapping the original protein ID to our pathogen proteomes was not enough; we also had to assign the antigenicity to the corresponding peptides within each antigenic protein. However, while our peptides were of fixed length, the curated antigenic sequences varied in size. For this reason, we developed our own mapping method that we called ‘kmer expansion’, which works by marking as antigenic any peptide that shared a kmer of at least 8 amino acids with a curated antigenic sequence for that same protein. The amount of total and antigenic peptides, before and after the ‘kmer expansion’, are listed in **Table 3**.

**Table 3:** Amount of antigenic proteins and peptides for each species.

Species	Group	Proteins			Peptides		
		Total	Antigenic		Total	Antigenic	
			Original	After BLAST		Original	After kmer expansion
B. burgdorferi	Gram -	1,390	137	152	386,683	117	863
B. melitensis	Gram -	3,178	13	13	–	–	–
C. burnetii	Gram -	1,853	102	104	–	–	–
E. coli	Gram -	4,778	7	7	1,428,744	9	158
F. tularensis	Gram -	1,556	27	27	–	–	–
L. interrogans	Gram -	3,683	10	10	1,113,309	19	342
P. gingivalis	Gram -	1,881	10	11	626,536	165	1181
M. leprae	Gram +	1,605	7	8	515,942	76	633
M. tuberculosis	Gram +	3,940	81	89	1,268,272	416	4,369
S. aureus	Gram +	2,607	16	16	758,970	55	575
S. pyogenes	Gram +	1,690	13	13	491,619	263	985
L. braziliensis	Eukaryote	8,084	8	12	4,964,396	14	182
P. falciparum	Eukaryote	5,337	106	131	4,009,580	562	9,120
T. gondii	Eukaryote	8,322	15	16	6,535,220	94	457
T. cruzi	Eukaryote	21,170	242	2,480	10,408,841	4,025	7,317

This table shows the amount of antigenic proteins and sequences extracted from bibliography and the final amount after processing. For proteins, BLAST was used to also tag as antigenic other proteins of the same species that were similar to the antigenic ones. For peptides, a custom mapping method named ‘kmer expansion’ was used to tag peptides as antigenic based on the antigenic sequences in bibliography (see Methods). We did not have information at peptide level for three of the species.

In the case of *Onchocerca volvulus*, the method we used to derive antigenic proteins and peptides was based on experimental proteome-wide data on antibody-binding to short peptides (41). We followed the same rules used by these authors to find the peptides they called ‘immunoreactive’. Because these peptides had lengths from 8 to 15 amino acids, we assigned as antigenic any neighboring peptides that shared at least 8 amino acids with them (this is stricter that using our ‘kmer expansion’ strategy because it limits the antigenicity to that section of the protein).

### Clustering by Sequence Similarity

It is common practice in the literature to report antigenicity for a single or a few reference proteins or accession numbers. This information is then passed on to databases such as IEDB (25, 26). Nevertheless, when dealing with complete proteomes, there are usually other paralogs with high sequence similarity to those labeled as antigenic. Since they have similar sequences, these proteins would then have similar properties which would likely result in similar outputs when running the predictors. However, because only one of those proteins is labeled as antigenic, this would hinder the learning capabilities of any models trained or tested with these data.

To improve the learning process of APRANK, and to account for unlabeled proteins, we calculated sequence similarity for all proteins in the 15 analyzed proteomes using blastp from the NCBI BLAST suite (42) (console call in **Supplementary Table S2**). We then wanted to filter the BLAST output keeping only the good matches, which meant selecting a similarity threshold. After analyzing different matches, we arrived at a sensible compromise: trying to be as strict as possible without losing much data. For this we kept matches with a percentage of identical amino acids (pident) of at least 0.75, an expected value (evalue) less than or equal to 1 x 10^-12^ and a match length of at least half of the length of the shortest protein in the match.

Using these matches, we created a distance matrix where *distance* = 1 – *pident* and applied a single-linkage hierarchical clustering method. We then cut this tree using a cutoff of 0.25 (1 - *pidentThreshold*), resulting in a set of clusters of similar proteins.

For the species-specific models, proteins in a given cluster were kept together in the training process, meaning they would all be either in the training set or in the test set.

For the generic models, any protein in the training set which belonged to a cluster with at least one other antigenic protein was also tagged as antigenic, even across species (obviously excluding the species being tested). As for the test set in the generic models, this would also occur, but only inside that same species. The amount of total and antigenic proteins, before and after using BLAST to find similar proteins inside each species, can be seen in **Table 3**.

### Data Normalization

Each predictor used by APRANK varied on how they returned their values. Not only they had different value ranges, but while some of them returned their values per protein, others did so per peptide, kmer, or amino acid. For this reason, we needed to parse and normalize all outputs before feeding their data into our models.

Values returned by each predictor were normalized to fit a numeric range between 0 and 1. Different methods were used to parse and normalize the data for each combination of predictor and model, ranging from linear or sigmoid normalizations to a simple binary indicator of presence or absence of a given feature (such as signal peptide). The methods used to normalize the output for each predictor were the result of analyzing the distribution and spread of these outputs across all of our species for each predictor individually, coupled with biological knowledge of what each predictor was analyzing. Some predictors that returned information exclusively at protein level were not used in the peptide models. The detailed steps on how to parse and normalize the output of each predictor for the protein and the peptide models are described in **Supplementary Table S3** and the formulas used for these operations can be found in **Supplementary Article S1**. Furthermore, this is also documented in the code (software) available at Github (see *Availability*).

### Fitting the Species-Specific Models

**Figure 1:**
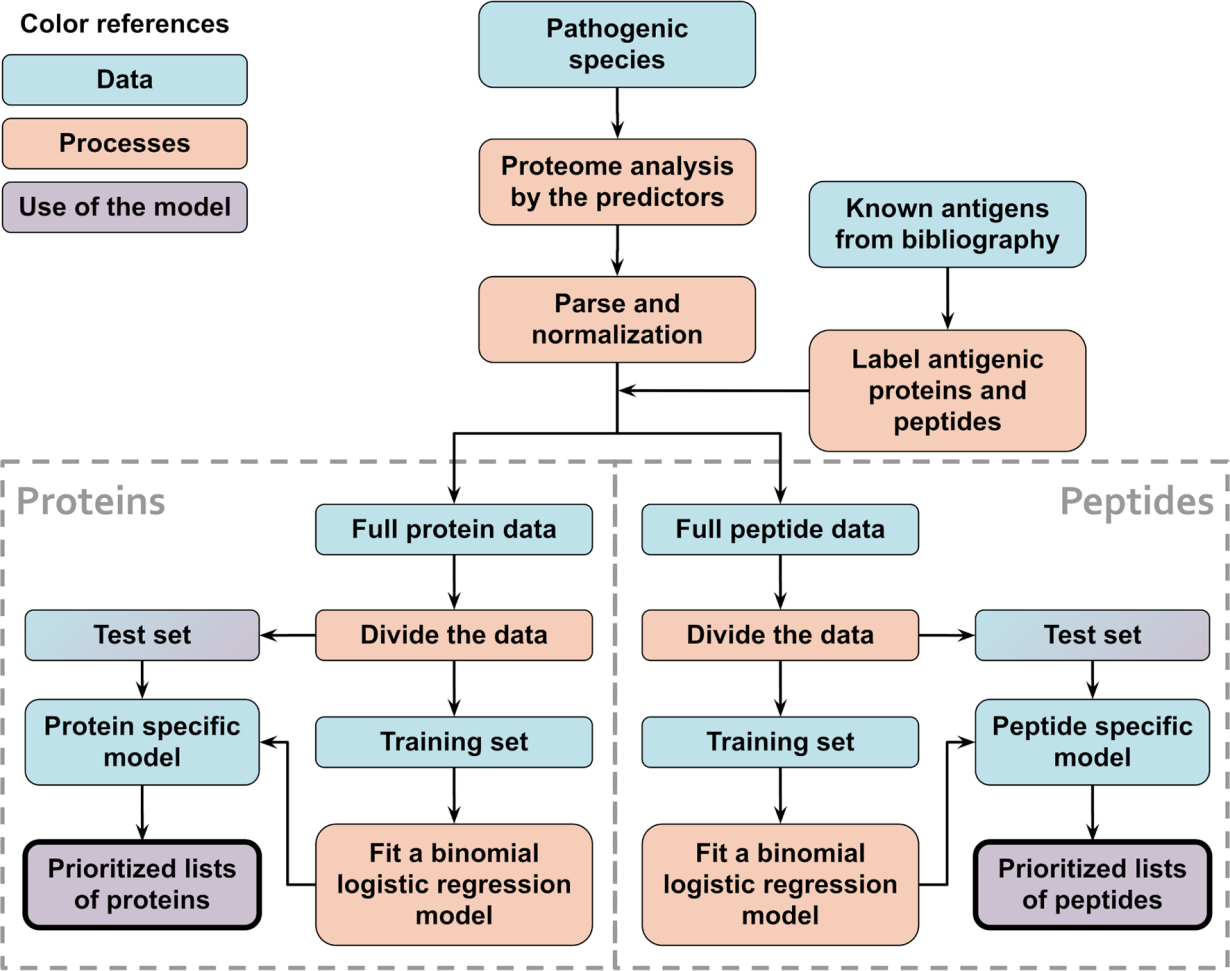
Schematic flowchart used to obtain APRANK’s species-specific models. With the aim of testing and tuning our method, training and prioritization was performed for both proteins and peptides using data from a single proteome of interest. This process was repeated for all of our 15 species.

Species-specific models were created to test the method and compare between balanced and unbalanced training sets. In this case, separate models were created for each species, using only train/test data from that organism alone. A schematic flowchart showing the logic of this procedure is shown in **Figure 1**.

To fit each protein species-specific model, clusters for that species were divided in training and test sets in a 1:1 ratio due to the low number of recorded antigens for some species. For this same reason, the training set was balanced with ROSE (43), generating an artificial training set with a similar number of antigenic and non-antigenic artificial proteins. This process, as well as all other described below, was repeated 50 times by re-sampling the clusters in the training and test sets.

A binomial logistic regression model was fitted for both the balanced and the unbalanced training sets using the generalized linear models in R (function glm). We chose this model for two reasons: because it allowed us to see a direct relationship between the models and our predictors *via* the coefficients of the model, and because it was not as affected as other more complex models by the existence of false negatives (which we knew existed because they were the novel antigens we wanted to find). Once the balanced and the unbalanced protein models were trained, we used them to predict the scores for the test set. The performance for each model, measured by the area under the ROC curve (AUC), was then calculated using the R package pROC (44). Additionally, two pseudo random set of scores were created by shuffling the scores achieved by both models. These random protein models were used to test if the performance of our models differed significantly from a random prediction.

For the peptide species-specific models, we divided the peptides into training and test sets by simply following the division of the proteins clusters, meaning that if a protein was in the training set for the protein model, its peptides would be in the training set for the peptide model for that iteration. The models were fitted and random scores calculated in a similar manner to the protein models. However, when we attempted to calculate the performance of the peptide models, our test set was too large to calculate performance based on AUC values in a reasonable time. We decided then to sample a subset of 50,000 peptides from the test set in a pseudo-random manner, making sure that the positive peptides were found in the subset and that the fraction of positive vs indeterminate/negative antigens was similar to the one in the test set (but never below 1% unless we ran out of antigens). All AUC values for the different peptide models were calculated using the same subset, and this process was repeated 5 times in each iteration, changing the subset each time.

Once all iterations were finished, we compared the AUCs obtained by the balanced and unbalanced versions of the protein and peptide species-specific models using a Student’s t-test. Another set of t-tests were used to analyze the difference between each of those models and their relative random model. If the model had a significantly higher AUC than the corresponding random model, we considered the model achieved a successful prediction (p < 0.05).

### Creating the Generic Models

The generic (pan-species) models are the actual models used by APRANK. The objective of these models is to generalize predictions of antigenic proteins or peptides for new species (not used for training APRANK). In a broad sense, they have to learn what makes a protein or a peptide antigenic. We achieved this by training the models with a large set of antigenic proteins and peptides from 15 different species, including gram-negative bacteria, gram-positive bacteria and eukaryotic protozoans.

**Figure 2:**
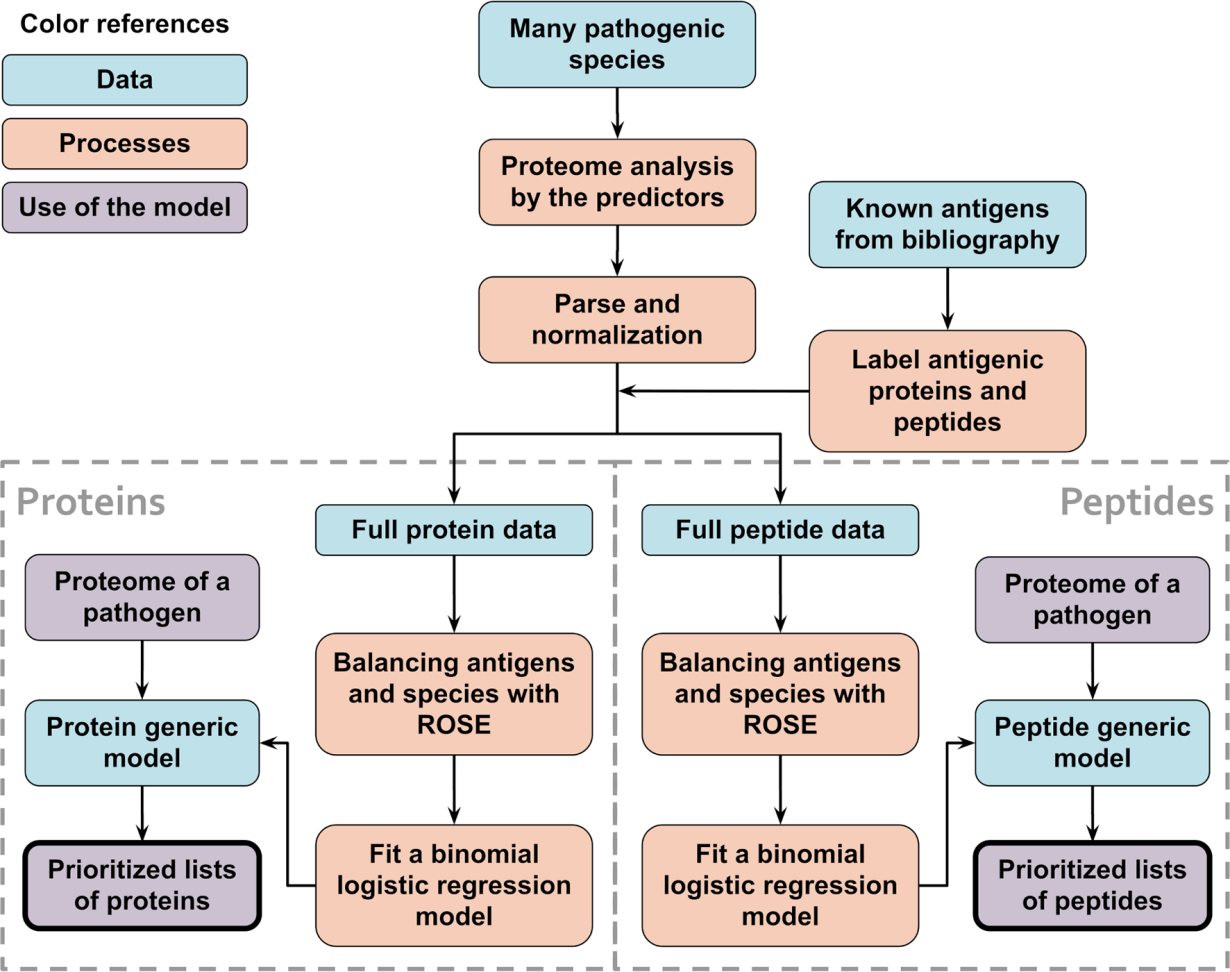
Schematic flowchart used to obtain APRANK’s generic models. With the aim of creating a set of models that could make predictions for a wide range of species, training and prioritization was performed for both proteins and peptides using combined data from all of our 15 species. When testing the generic models, leave-one-out models were used, where 14 species were used to train the models and the 15th species to test them. This process was repeated for all of our 15 species.

To create the protein generic models, we used ROSE (43) to make a balanced training set of 3,000 proteins for each species and then merged all those balanced training sets together. With these data, a linear model was created following the same steps as for the species-specific models. Next, these models were used to predict the scores for the species being analyzed and the performance of the prediction was calculated the same way as for the species-specific protein models. A schematic visualization of this procedure is shown in **Figure 2**.

We created the peptide generic models in a similar manner, with balanced training sets from each of the species that contained 100,000 peptides each. In addition to the regular score calculated by using the model to predict the antigenicity of the test data, we also calculated a combined score, which is simply the mean of the peptide score and the corresponding protein score. The performance of the peptide generic models was calculated the same way as for the species-specific peptide models.

When testing these generic models, we created temporary leave-one-out generic models, where we used 14 of the species to generate the protein and peptide models, and then tested the models in the 15th species. We then generated the final protein and peptide generic models using all 15 species and tested them by predicting antigenicity in *Onchocerca volvulus*, a novel species for APRANK, with experimental proteome-wide data (41).

### Comparative Performance

To discard the possibility that our model was simply detecting sequence similarity, we created a ‘BLAST model’, where we assigned to each protein a score based solely on how similar they were to a known antigenic protein from another organism. The score used was –*log*_10_(*evalue*) and then performance was calculated for each species.

We also wanted to make sure our model was combining information from several predictors. To rule out that performance was mainly driven by one predictor, we compared our prediction capabilities against the individual predictor with best AUC, which was BepiPred 1.0. To do this, the BepiPred score for each protein and peptide was obtained from the individual amino acid scores following the same steps we used for APRANK as detailed in **Supplementary Table S3**, but without normalizing it. The AUCs for the BepiPred peptide scores were calculated the same way as for the peptide species-specific models.

### Availability

The code for running or modifying APRANK, as well as the FASTA files of the different organisms and the detailed lists of antigens used in training, is available at the GitHub repository located at (https://github.com/trypanosomatics/aprank) (45), released under a BSD 2-Clause ‘Simplified License’, which is a permissive free software license. Because we do not have the rights to distribute software for some predictors, the repository also holds documentation on how to find, install and configure these dependencies (users are responsible for obtaining the corresponding licenses or permissions for some required predictors).

Because of their size, the trained generic models for proteins and peptides were deposited in Dryad under DOI:10.5061/dryad.zcrjdfnb1 (https://doi.org/10.5061/dryad.zcrjdfnb1).

## Results

Our aim in this work was to develop a computational method and associated pipeline capable of prioritizing candidate antigenic proteins and antigenic determinants (epitopes) from complete pathogen proteomes for downstream experimental evaluation. We have previously shown for *Trypanosoma cruzi* (Chagas Disease) that different criteria can be integrated and exploited in a computational strategy to further guide the process of diagnostic peptide discovery (11). Here we extend this work to other human pathogens and improve the way in which features are weighted, hence providing a tool for the prioritization of candidate linear B-cell epitopes for a wide range of pathogens.

### Species and Features

We selected human pathogens from a phylogenetically diverse set of taxa with experimentally validated antigen and/or epitope data to train and test our method. This included gram negative bacteria, gram positive bacteria and eukaryotic protozoans. We did not include viruses in this version of APRANK because they have small proteomes that are already amenable to experimental experimentation (e.g. their full-proteomes fit on standard low-density protein or peptide microarrays). The species selected to train APRANK and the diseases they cause are shown in **Table 1**.

We obtained the proteomes of these species (see *Methods*) and split each protein into peptides of 15 residues. Once this was done, we used information from the immune epitope database (IEDB) along with manually extracted information from several papers to tag each protein and peptide as antigenic or non-antigenic. The ‘non-antigenic’ tag in this paper should be understood in the sense of proteins with no prior information on their antigenicity. The amount of total and antigenic proteins and peptides can be seen in **Table 3**.

To develop a tool that can help identify candidate antigenic proteins and peptides, we used several predictors that focused on different properties of the proteins (**Table 2**). On a broad sense, these predictors assess: the antigenicity and/or immunogenicity of proteins (15, 17); the structural and post-translational features that can be predicted from the protein sequence, some of which may suggest the protein enters the secretory route or is anchored at the membrane (18, 21, 22); the presence of internal tandem repeats in proteins, which have been described to modulate immunogenicity of proteins (24) together with other structural features such as the presence of intrinsically unstructured or exposed regions in proteins which may affect their presentation in the context of an immune response (20, 23, 46, 47).

We have also implemented in APRANK a number of custom Perl and R scripts that measure sequence similarity between each pathogen protein and the human host (CrossReactivity), or itself (SelfSimilarity). The idea behind these measurements was to obtain additional information on highly conserved sequences that may result in e.g. potential lack of immune response (tolerance) if the pathogen sequence is highly similar to a human protein; or cross-reactivity of antigens and epitopes in other proteins from the same pathogen (self-similarity). These predictors provide information on desirable and undesirable properties that then need to be weighted accordingly to achieve good performance at the task of antigen and epitope prediction.

We then calculated which predictors were better in discerning antigenic proteins by themselves. For this we analyzed the distribution of the predictors’ parsed outputs looking for cases where the mean for the antigens were significantly different than the mean for the whole proteome (see **Supplementary Table S4**). BepiPred, NetMHCIIpan, NetSurfp and SignalP showed the best results, separating the means of antigens and non-antigens in over 9 organisms. Other predictors, such as Molecular Weight, were also able to achieve this, but weren’t consistent among species in who had the largest mean. This meant that they could prove useful when making species-specific models, but not when creating generic models, which was our true goal. Looking at the table, there was an argument to be made for removing some predictors from the model, but we decided to keep them for now and let the model likely assign lower coefficients to them (as can be seen in the generic leave-one-out models in **Supplementary Figure S3**). We talk more about these predictors in the Discussion.

### Testing APRANK and ROSE on Species-Specific Models

Species-specific models were created to test the method and to compare between unbalanced training sets and training sets balanced using ROSE (see *Methods*). As the name implies, these models worked with only one species at a time, using a fraction of its proteins to predict antigenicity for the rest. After running the predictors for all proteins in the selected genome, we parsed and processed the different outputs and applied a normalization process to have them in a common scale.

We needed to divide our data into training and test sets. Often, training sets represent ~ 80% of the data; however, in our case some species had a low number of validated antigens (see **Table 3**), which meant that choosing a 80/20 training/test set split would result in test sets having only a few antigenic proteins. This kind of imbalance tends to compromise the training process, making the model to focus on the prevalent class (non-antigenic) and ignore the rare class (antigenic) (48). For this reason, when training a model using data from a single species, we chose to split the training and test set 50/50, re-sampling proteins and peptides multiple times (see *Methods*). To improve the training process, we also used ROSE to balance our training sets, which works by generating artificial balanced samples from the existing classes, according to a smoothed bootstrap approach (43). Furthermore, we used the similarity-based clustering of sequences to avoid placing highly similar sequences into both training and test sets.

**Figure 3:**
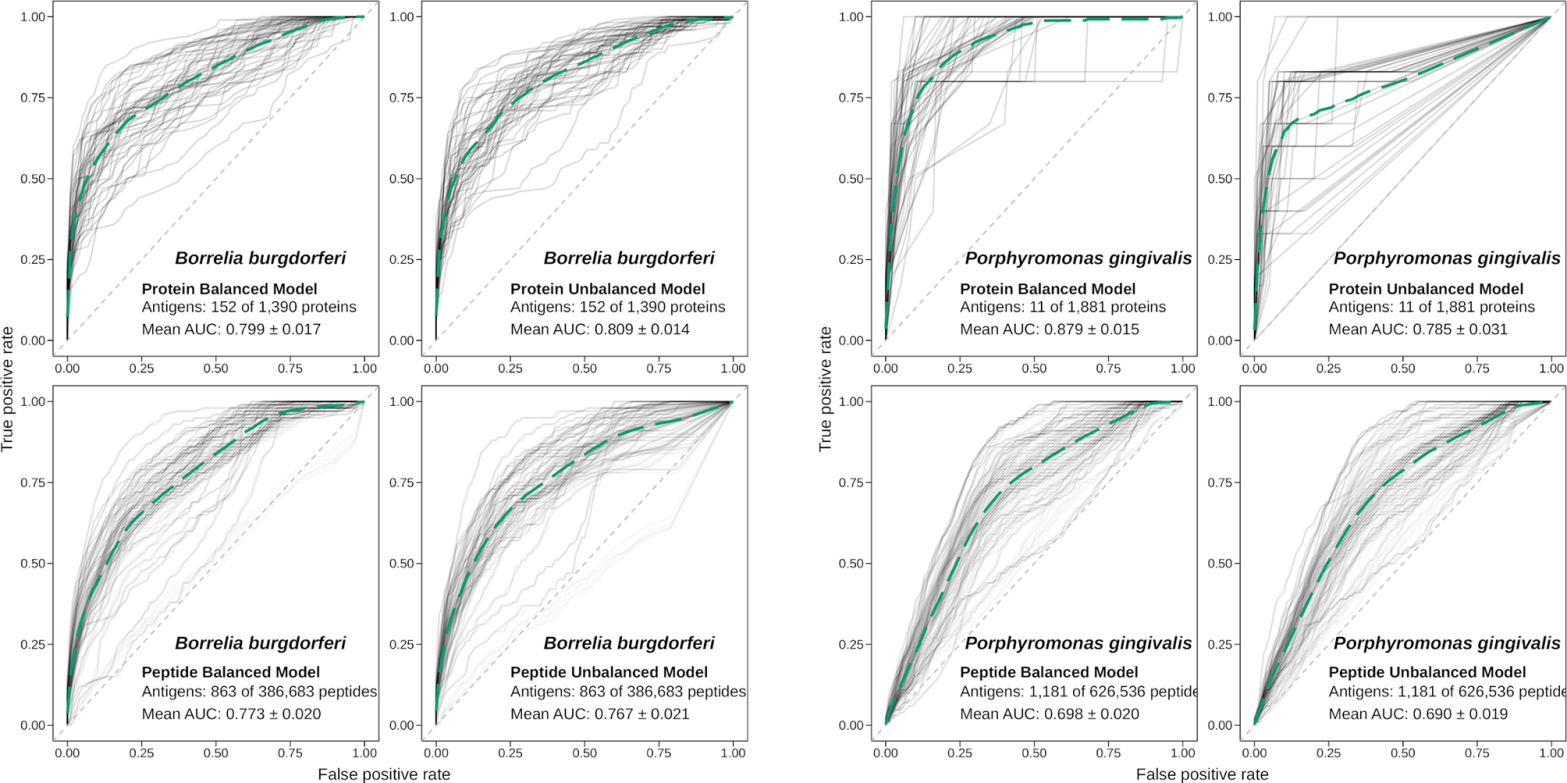
Performance of APRANK training using balanced or unbalanced data. Performance of APRANK’s species-specific models for *B. burgdorferi* and *P. gingivalis*. ROC curves for each iteration of training and testing are shown in light gray, and the average curves are shown in green (dashed lines).

We used these balanced training sets to fit a binomial logistic regression model, resulting in one model for proteins and one for peptides. These models, which we denominated *species-specific models*, were then used to predict the antigenicity of their respective test sets. The performance of APRANK was assessed by measuring the area under the ROC curve (AUC), using known antigens and epitopes in the protein and peptide test sets. This whole process was repeated 50 times, re-sampling which proteins were in the training set and which in the test set. A final APRANK AUC score for each species was calculated as the mean of all AUC scores for these iterations (see **Figure 3**). To assess the effect of balancing the data on our models using ROSE, we also assessed the performance of APRANK repeating the procedure described above using the unbalanced training sets instead, resulting in a set of AUC scores corresponding to species-specific models trained with unbalanced data.

These calculations were done for each of the 15 species, although for 3 of them there was no antigenicity information at the peptide level, and only protein models were calculated. The results are presented in **Table 4**. Our testing showed that APRANK was able to predict antigenicity for proteins and peptides in most cases, with good performance. The only species that did not have a successful prediction were *E. coli* for the protein model and *M*. *tuberculosis* and *S. aureus* for the peptide model. In these cases, the final AUC corresponding to the species-specific model was not significantly different than a random prediction. As for the balancing of the data using ROSE, it seemed to have mostly positive or neutral effects in the predicting capabilities of our models, which meant we could safely use it in training our pan-species models.

**Table 4:** Prediction results for the specific models.

Species	Proteins			Peptides		
	BTR	Trained with unbalanced data	Trained with balanced data	BTR	Trained with unbalanced data	Trained with balanced data
		Mean AUC	Mean AUC		Mean AUC	Mean AUC
B. burgdorferi	Yes	0.809 ± 0.014	0.799 ± 0.017	Yes	0.767 ± 0.021	0.773 ± 0.020
B. melitensis	Yes	0.710 ± 0.037	0.700 ± 0.033	–	–	–
C. burnetii	Yes	0.611 ± 0.011	0.620 ± 0.010	–	–	–
E. coli	**No**	0.511 ± 0.034	0.515 ± 0.039	Yes	0.584 ± 0.056	0.633 ± 0.047
F. tularensis	Yes	0.783 ± 0.018	**0.807 ± 0.014** ^*^	–	–	–
L. interrogans	Yes	0.827 ± 0.033	0.867 ± 0.023	Yes	0.559 ± 0.015	0.565 ± 0.011
P. gingivalis	Yes	0.785 ± 0.031	**0.879 ± 0.015** ^***^	Yes	0.690 ± 0.019	0.698 ± 0.020
M. leprae	Yes	0.633 ± 0.018	0.652 ± 0.018	Yes	0.557 ± 0.029	0.585 ± 0.023
M. tuberculosis	Yes	0.635 ± 0.010	0.647 ± 0.011	**No**	0.508 ± 0.010	0.502 ± 0.010
S. aureus	Yes	0.765 ± 0.032	0.772 ± 0.023	**No**	0.438 ± 0.054	0.420 ± 0.057
S. pyogenes	Yes	0.884 ± 0.039	**0.984 ± 0.003** ^***^	Yes	0.832 ± 0.021	0.844 ± 0.019
L. braziliensis	Yes	**0.719 ± 0.021** ^**^	0.673 ± 0.020	Yes	0.778 ± 0.029	**0.867 ± 0.025** ^***^
P. falciparum	Yes	0.821 ± 0.009	0.826 ± 0.007	Yes	0.758 ± 0.016	**0.779 ± 0.012** ^*^
T. gondii	Yes	0.656 ± 0.032	**0.744 ± 0.032** ^***^	Yes	**0.646 ± 0.035** ^**^	0.584 ± 0.020
T. cruzi	Yes	0.803 ± 0.029	**0.850 ± 0.022** ^*^	Yes	0.838 ± 0.019	0.854 ± 0.016

The prediction was considered to be successful if it was significantly Better Than a Random set of scores (BTR). Each specific model was calculated 50 times using different, but overlapping, subsets of data as training and test sets. In bold we show the model with the significantly higher AUC when comparing training with unbalanced or balanced data (Student’s t-test, *< 0.05, **< 0.01, ***< 0.001).

### Development of APRANK as a Pan-Species Ranker of Antigens and Epitopes

In the previous section we used protein and peptide data from a given pathogen species to train models that successfully predicted antigenicity for that same organism; however, our end goal was to have models that were able to predict protein and peptide antigenicity for any pathogen. To achieve this, we created models trained with all species, which we called *protein generic models* and *peptide generic models*.

For these models, we used ROSE (43) to generate similar sized partitions of balanced data for each of the species, and then we merged these data and fitted two binomial logistic regression models, one for proteins and one for peptides. When using the models to predict the peptide antigenicity scores, we also analyzed the predicting capabilities of what we called the *combined score*, which was a combination of the protein and peptide scores for a given peptide.

To validate these models we performed a leave-one-out cross-validation method (LOOCV), hence creating 15 different protein generic models, each time leaving out one species (which was the one being used as test set). For the peptide generic models we followed a similar route, but we ended up with 12 models due to the lack of antigenicity information at peptide level for 3 of the 15 species.

The performance results for these models are presented in **Table 5**. The generic protein models were successful in predicting antigenicity for all species, and similar results were obtained also at the peptide level, achieving successful predictions even for *E. coli*, *M. tuberculosis* and *S. aureus*, which were the three species where the species-specific models performed poorly before. This observation suggests that performance is related to the amount and diversity of recorded antigens. As for the performance of these generic models, the observed AUC scores obtained similar values to the ones obtained in the species-specific models trained with balanced data, indicating that while these generic models did not have information about the species being tested, the data obtained from all the other 14 species was enough to learn the generic rules that made a protein antigenic. The scores produced by APRANK for each protein and the best peptides for each of these 15 species can be found in the Supplementary Data, deposited in Dryad under DOI:10.5061/dryad.zcrjdfnb1.

**Table 5:** Prediction results for the leave-one-out generic models.

Species	Proteins		Peptides			
	BTR	LOO model	BTR	LOO model	LOO model + protein scores	Combined score relative AUC gain
B. burgdorferi	Yes	0.786	Yes	0.768	0.950	**23.60%**
B. melitensis	Yes	0.774	–	–	–	–
C. burnetii	Yes	0.620	–	–	–	–
E. coli	Yes	0.754	Yes	0.742	0.780	**5.12%**
F. tularensis	Yes	0.698	–	–	–	–
L. interrogans	Yes	0.947	Yes	0.679	0.948	**39.57%**
P. gingivalis	Yes	0.854	Yes	0.665	0.871	**30.91%**
M. leprae	Yes	0.758	Yes	0.692	0.731	**5.68%**
M. tuberculosis	Yes	0.702	Yes	0.586	0.711	**21.17%**
S. aureus	Yes	0.737	Yes	0.752	0.790	**5.03%**
S. pyogenes	Yes	0.983	Yes	0.838	0.970	**15.81%**
L. braziliensis	Yes	0.709	Yes	0.946	0.878	**-7.20%**
P. falciparum	Yes	0.807	Yes	0.748	0.835	**11.66%**
T. gondii	Yes	0.837	Yes	0.583	0.720	**23.51%**
T. cruzi	Yes	0.867	Yes	0.843	0.857	1.58%

The prediction was considered successful if it was significantly Better Than a Random set of scores (BTR). For peptides, we show both the performance of the model alone, and the performance obtained by combining the protein and peptide scores. In bold we show any difference greater than 5% between the peptide score and the combined score for a given species. LOO Model, Leave-One-Out Model.

We also reached similar conclusions when comparing the coefficients obtained in the different protein models. In the case of individual (species-specific) models, coefficients were less robust across iterations when there were few positive cases, and more robust with larger validated training examples, as expected (see **Supplementary Figures S1** and **S2**). For the pan-species models, we found the coefficients to be very robust across all 15 models, indicating that the different leave-one-out generic models reached a similar conclusion on what makes a protein ‘antigenic’ (see **Supplementary Figure S3**). This reinforces the idea that better performance is the result of more extensive training with diverse positive and negative examples.

### Using APRANK to Obtain Antigen-Enriched Sets

Our generic models allowed us to rank proteins and peptides in a given species based on a model trained from other pathogens. Now, we wanted to use these scores to select a subset of proteins or peptides with an increased chance of being antigenic when compared to the whole proteome.

**Figure 4:**
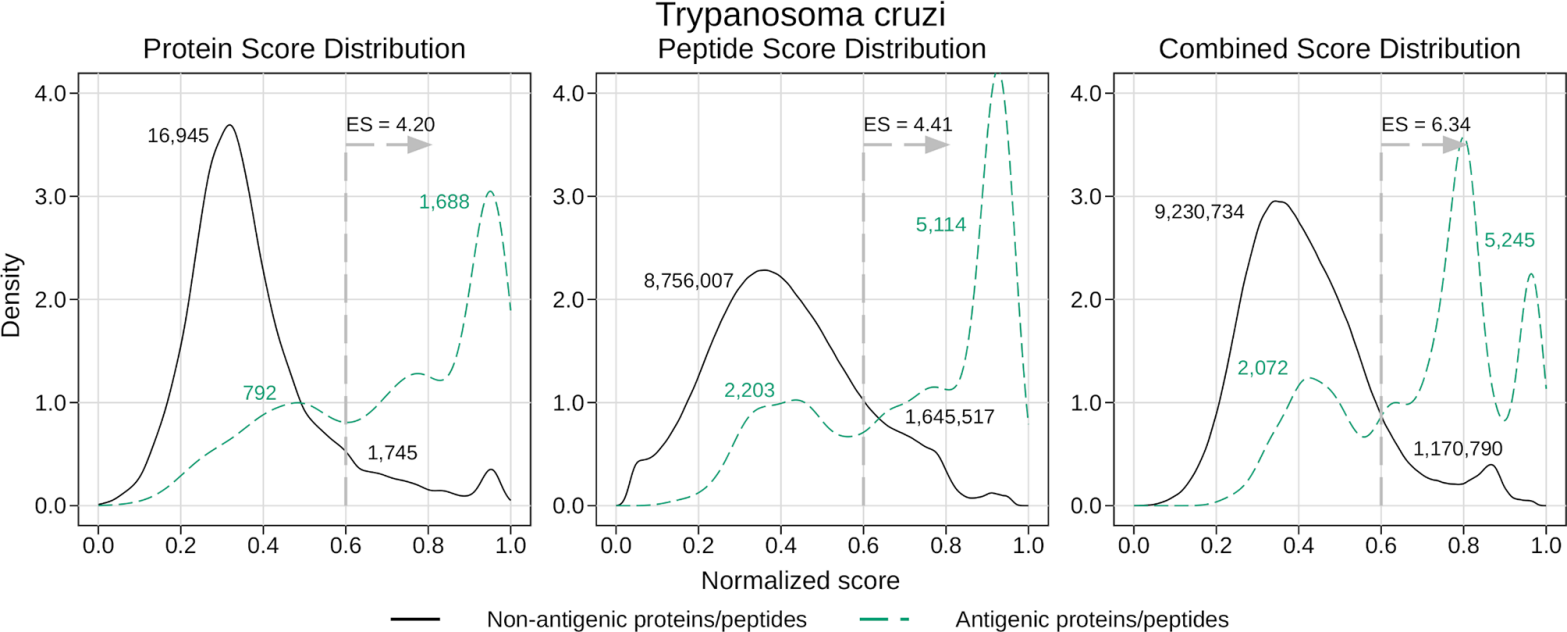
Density analysis for the antigenicity scores of *T. cruzi*. Plots were obtained by analyzing the proteome of *T. cruzi* with the leave-one-out generic models, and then distinguishing between antigens and non-antigens. The figure shows the enrichment score obtained by keeping only the proteins and peptides with a score greater than 0.6, as well as the amount of antigens and non-antigens that would be inside or outside that subset.

For this, we focused on *T. cruzi*, as this was the species with the largest number of recorded antigens within our collection. To obtain fair antigenicity scores for this protein we used the corresponding leave-one-out models created when testing the generic models. We analyzed the distribution of the normalized scores returned by these models, distinguishing between antigenic and non-antigenic proteins and peptides (see **Figure 4**). As was expected, the peak of the scores for the antigens is found to the right of the one for the non-antigens, indicating that the average score is higher for the antigenic proteins and peptides. Also, the amount of overlapping can be related to the corresponding AUC, where the higher the AUC, the less the overlapping.

Once we had our score distributions, we used them to select an antigen-enriched subset of proteins and peptides. This could be done in one of two ways: either by setting a score threshold or by simply selecting a fixed number of proteins and peptides within the top scores. After analyzing the distribution of score values, we decided to use the first option and selected those proteins and peptides with a normalized score of at least 0.6. We next calculated what we called *enrichment score* (ES), which was the proportion of antigens in the selected subset relative to the proportion of antigens in the whole proteome (for example, ES = 2 meant you were twice as likely to find an antigen in the subset than in the whole proteome, or in a random subset). In **Figure 4** we show the enrichment scores for the different normalized scores and the number of proteins and peptides that fall inside or outside those subsets. While the subsets were usually a small fraction of the whole proteome (close to 10% in most cases), this represents a 4 – 6 fold increase in the chances of finding antigens in those subsets.

As an example, suppose a microarray with a capacity of 200,000 unique peptides. Based on the current antigenic data we possess, a random sampling of the *T. cruzi* proteome would lead to the inclusion of ~ 140 antigenic peptides in that microarray. However, using APRANK to select the top 200,000 peptides with the highest normalized combined score, we would end up including almost 1,600 antigenic peptides in the array (an enrichment score of 11.35). This demonstrates the utility of tools like APRANK for selection of antigenic peptides for screening platforms.

### Assessing the Validity of the Computational Method

Now that we had a working pan-species model, we validated the performance achieved by APRANK. For this, we first assessed that the performance was the result of combining information from different predictors, and not from just one or a few of them.

To do this, we selected the predictors that managed to consistently discern antigenic proteins (see **Supplementary Table S4**) and we calculated the area under the ROC curve (AUC) for both known proteins and known peptides in each case (not shown). We found that the predictor with best solo predicting capabilities was BepiPred 1.0. We then compared BepiPred’s predictions against APRANK’s for both the protein and peptide generic models for each species. This is presented in **Table 6**.

**Table 6:** Comparison between APRANK and the predictor with highest solo AUC (BepiPred 1.0).

Species	Proteins			Peptides		
	BepiPred score AUC	APRANK score AUC	APRANK relative AUC gain	BepiPred score AUC	APRANK score AUC	APRANK relative AUC gain
B. burgdorferi	0.729	**0.786**	**7.94%**	0.796	0.768	-3.46%
B. melitensis	0.710	**0.774**	**8.93%**	–	–	–
C. burnetii	0.558	**0.620**	**11.13%**	–	–	–
E. coli	0.587	**0.754**	**28.39%**	0.662	**0.742**	**12.21%**
F. tularensis	0.570	**0.698**	**22.40%**	–	–	–
L. interrogans	0.839	**0.947**	**12.87%**	0.676	0.679	0.42%
P. gingivalis	0.852	0.854	0.25%	0.674	0.665	-1.36%
M. leprae	**0.868**	0.758	**-12.67%**	0.689	0.692	0.51%
M. tuberculosis	0.666	**0.702**	**5.29%**	0.561	0.586	4.58%
S. aureus	0.723	0.737	1.86%	0.767	0.752	-1.93%
S. pyogenes	0.970	0.983	1.33%	0.800	0.838	4.73%
L. braziliensis	0.549	**0.709**	**29.00%**	0.905	0.946	4.48%
P. falciparum	0.793	0.807	1.84%	0.642	**0.748**	**16.42%**
T. gondii	0.579	**0.837**	**44.59%**	0.584	0.583	-0.21%
T. cruzi	0.814	**0.867**	**6.54%**	0.819	0.843	3.03%

The relative AUC gain shows the increase or decrease of the AUC obtained by our method relative to the one obtained by BepiPred. Differences greater than 5% are shown in bold.

We focused on those cases where the AUC changed at least 5% between BepiPred 1.0 and APRANK’s generic models. APRANK showed increased predicting capabilities for 11 out of the 15 analyzed proteomes at the level of complete proteins and/or peptides, while showing a decrease in performance only in *M. leprae* at protein level. These results provide validation support to the approach built into APRANK by combining information from many predictors.

As an additional test, we also assessed the performance of APRANK after removing BepiPred 1.0 predictions from our model. This can be seen in **Supplementary Table S5**. In this simulation we observed that even without BepiPred 1.0, our model reached similar predicting capabilities in most cases, hence suggesting that other predictors and features included in APRANK were able to replace BepiPred when training the model (this is further discussed in the *Conclusions*).

To ensure that our model was doing more than simply detecting sequence similarity, we also compared our performance against a ‘BLAST model’, meaning a model that was based solely on how similar a given protein was to a known antigenic protein. The comparison between the performance of this model and APRANK can be seen in **Supplementary Table S6**. As expected, APRANK achieved a larger AUC for most for the species; however we observed that for *M. leprae* and *L. braziliensis* the ‘BLAST model’ actually resulted in a better prediction. This may be explained because these were species with a small number of validated antigens (test cases) and a with high similarity to other of our selected species. To test this, we repeated this analysis for these two species, but now we removed from the BLAST model the species that were most similar to the one being analyzed (see bottom rows in **Table S6**). The performance under these altered conditions indeed resulted in significantly lower AUCs, matching or falling behind APRANK.

### Applying Our Method on a Novel Species

To truly validate APRANK, we wanted to test the method on a new species that was not included in our initial training and that had an extensive amount of information on the antigenicity of its proteins and peptides. For this, we searched for publications containing proteome-wide linear epitope screenings using high-density peptide microarrays and selected Lagatie et al. (41), a recent dataset produced by scanning the complete *Onchocerca volvulus* proteome with more than 800,000 short peptides (mostly 15mers). *Onchocerca volvulus* is a nematode and it is the causative agent of Onchocerciasis in humans (also called river blindness), a disease that is on the list of Neglected Tropical Diseases (NTDs) of the World Health Organization (49).

We obtained a list of antigens from *O. volvulus* following the same rules applied by the authors to find the peptides they called ‘immunoreactive’ (see Methods in Lagatie et al.), resulting in a set of almost 1,100 antigenic peptides. We tagged a protein as antigenic if it had at least one of these peptides; however, we also kept information on how many ‘immunoreactive’ peptides each protein had for later analysis. Once this was done, we also tagged as antigenic any neighboring peptide that shared at least 8 amino acids with one of these ‘immunoreactive’ peptides.

We next trained APRANK with all our 15 species and then used these models to predict the antigenicity scores for both the proteins and the peptides of *O. volvulus*. An AUC score was calculated for each prediction, comparing the score given by APRANK against the antigenic tag for each protein and peptide. We also calculated the enrichment scores for these scenarios using a score threshold of 0.6 in a similar way that we did for *T. cruzi*. The scores obtained by APRANK for each protein and the best peptides of *O. volvulus* can be found in the Supplementary Data, deposited in Dryad under DOI:10.5061/dryad.zcrjdfnb1.

**Figure 5:**
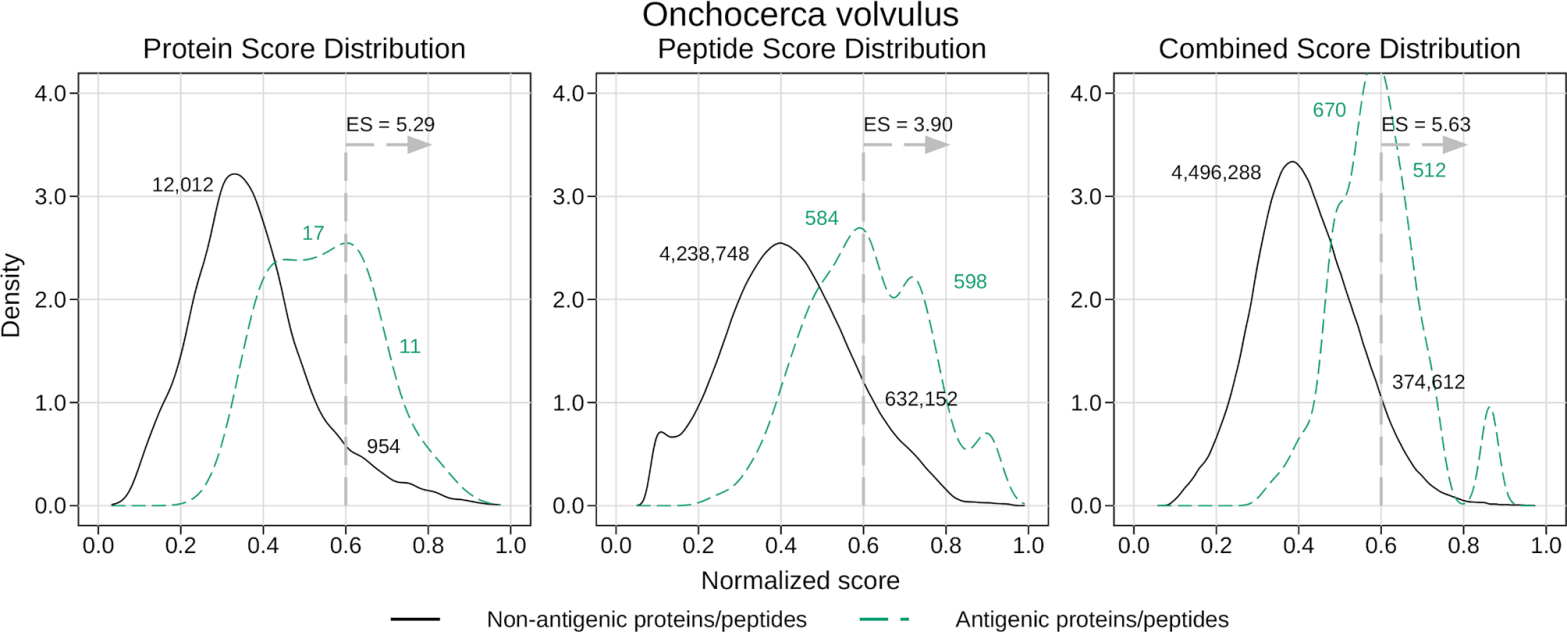
Density analysis for the antigenicity scores of *Onchocerca volvulus*. Plots were obtained by analyzing the proteome of *O. volvulus* with the final generic models, and then distinguishing between antigens and non-antigens. The figure shows the enrichment score obtained by keeping only the proteins and peptides with a score greater than 0.6, as well as the amount of antigens and non-antigens that would be inside or outside that subset. The plots correspond to the case where a protein was tagged as antigenic if it had at least 3 ‘immunoreactive’ peptides (see *Results*).

Our method was successful in predicting the antigenicity of proteins and peptides for *O. volvulus*, as shown in **Table 7**. We observed that if we were stricter when tagging a protein as antigenic, meaning requiring more ‘immunoreactive’ peptides, we obtained better performance. When considering as antigenic any protein with 1 ‘immunoreactive’ peptide we had an enrichment score of 2.28, whereas when we increased this requirement to 3 ‘immunoreactive’ peptides the enrichment score was 5.29 (see **Table 7** and **Figure 5**). Besides validating the performance of APRANK on a new pathogen, this suggests that either our method is better in predicting proteins with many antigenic regions, or that a single reactive peptide from a peptide array screening may provide only weak support for calling of antigens.

**Table 7:** Performance of APRANK on *Onchocerca volvulus*.

	Total	Score	#MIP	Antigenic	AUC	Antigens with score 0.6	Enrichment score for 0.6
Proteins	12,994	Protein score	1	886	0.677	150	2.28
			2	177	0.713	38	2.89
			3	28	0.828	11	5.29
Peptides	4,872,082	Peptide score	1	1,097 → 14,122	0.800	6,108	3.33
			2	397 → 4,498	0.798	1,995	3.42
			3	104 → 1,182	0.836	598	3.90
		Combined score	1	1,097 → 14,122	0.750	3,376	3.10
			2	397 → 4,498	0.774	1,342	3.88
			3	104 → 1,182	0.871	512	5.63

Proteins and peptides were tagged as antigenic based on the number of Minimum Immunoreactive Peptides (#MIP). For proteins, we considered as antigenic those with at least #MIP immunoreactive peptides. For peptides, we considered as antigenic any immunoreactive peptide found inside proteins with at least #MIP immunoreactive peptides. We show the number of antigenic peptides before and after spreading the antigenicity from the original immunoreactive peptides to their neighboring peptides (before → after). The rule to define an ‘immunoreactive peptide’ was extracted from Lagatie et al., 2017 (see Methods). The enrichment score represents the proportion of antigens in the selected subset relative to the proportion of antigens in the whole proteome.

For peptides, APRANK obtained an enrichment score of 3.33 – 3.90, also showing an additive effect when combined with the protein score, suggesting that these are effective in predicting antigenicity for *O. volvulus*. Similar to before, we tried being stricter and only considering antigenic peptides in proteins with at least 2 or 3 ‘immunoreactive’ peptides; however this did not seem to affect the predictive performance as much as for whole proteins.

### Applying Our Method on a Novel Dataset: Exploring Seroprevalence

As a final step, we also tested performance of APRANK on an additional dataset from *Plasmodium falciparum* that was not used as a source of validated antigens in our previous training. In Obiero et al., the authors analyzed the proteome of *P. falciparum* using a protein microarray which displayed ~ 91% of the proteome (50). But more importantly, they analyzed the individual antibody responses of 38 patients in controlled human malaria infections. Hence, they provided a rich set of information on seroprevalence for each analyzed protein.

As part of this validation we analyzed if the APRANK scores predicting antigenicity were in any way correlated with the observed seroprevalence. This seroprevalence data encompassed 4,768 unique genes, and was matched against APRANK protein scores for *P. falciparum*. To avoid the possibility of over-fitting, the APRANK scores were those obtained from the leave-one-out generic model trained in 14 species, but leaving out *P. falciparum*. This resulted in 4,343 proteins with information of both seroprevalence [from Obiero et al. (50)] and antigenicity score (from our work).

**Figure 6:**
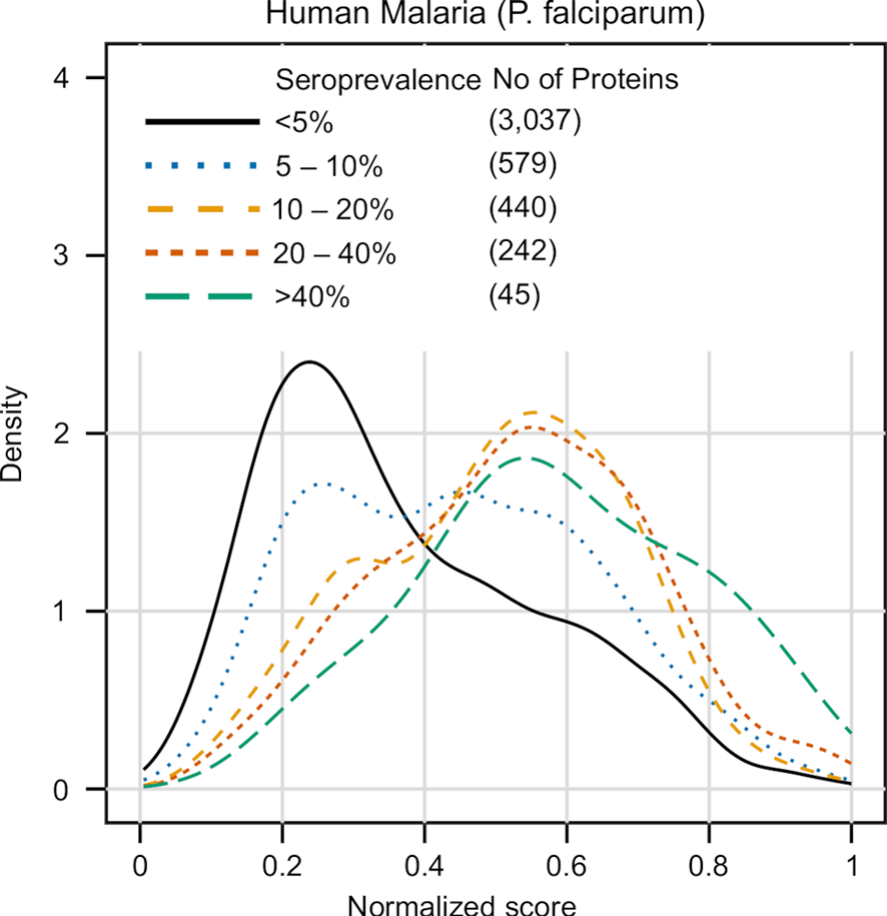
Validation of APRANK against antigens with known seroprevalence. Detailed information on the seroprevalence of *Plasmodium falciparum* proteins in cases of Human Malaria was obtained from (50) (n = 38). Proteins were clustered in different seroprevalence groups and matched against APRANK antigenicity scores (see *Results*).

The results of this analysis are summarized in **Figure 6**. Unlike previous cases where the proteins in the test set were put in binary classes (antigenic vs non-antigenic), here we divided the data in 5 groups, using seroprevalence cutoffs at the 5%, 10%, 20% and 40% levels. The distribution of APRANK scores for these groups showed that proteins with higher seroprevalence also had higher APRANK scores, and hence shift to the right of the plot. This was evident in the separation of the non-antigenic bulk of the proteome (< 5% seroprevalence) from those proteins that are in the 10% - 20% seroprevalence range, and also and importantly in the highly seroprevalente antigens (seroprevalence >= 40%), where the density of the peak shifts further towards higher scores. This was as well supported by the AUC prediction of these two groups, which was 0.660 for the 10% - 20% seroprevalence range and 0.740 for the >= 40% range. While further studies of this kind are necessary to explore the link between antigenicity and seroprevalence, these results further validate APRANK at the task of prioritizing antigenic proteins from complete proteomes.

## Discussion

We present APRANK, a novel method to prioritize and predict the best antigen candidates in a complete pathogen proteome. APRANK relies on a number of protein features that can be calculated for any protein sequence which are then integrated in a pan-species model. Our benchmarks show that by integrating multiple predictors, pooling antigen data from multiple species across a wide phylogenetic selection, and balancing training datasets, APRANK matches or outperforms a state-of-the-art predictor such as BepiPred 1.0 in most scenarios.

We have tested this integrative method using non-parametric ROC-curves and made an unbiased validation using two independent data sets (*O. volvulus* and *P*. *falciparum*) containing recent proteome-wide antigenicity data. In summary, we found APRANK to be successful in predicting antigenicity for all pathogen species tested, hence providing a new and improved method to obtain antigen-enriched protein and peptide subsets for a number of downstream applications.

### Conclusions: Looking Forward

While we are satisfied by APRANK’s performance, there are still ways to further improve it. The main issue we had when training our models is the current lack or sparsity of validated epitope and antigen information. Particularly, well validated non-antigenic sets are currently hard to find in the literature, forcing us to count as non-antigenic all proteins and peptides that do not currently have experimental evidence of antigenicity or were not tagged as antigenic in databases (which we know is hardly true). Obtaining validated data about non-antigenic proteins and peptides will improve the training of the models for future versions of APRANK.

We also observed that the performance of APRANK was not considerably affected by removing some individual features. This might indicate that, as we observed previously (11), each individual predictor contributes only slightly to the overall performance. Another alternative explanation is that there might be redundancy between some of the predictors. For example the features being used for training of BepiPred 1.0 HMMs [propensity scales for secondary structure preference and hydrophilicity of amino acid residues (15)] may overlap others used internally by some of the predictors in APRANK. Future versions of APRANK will review these overlaps, analyzing the pros and cons of adding novel predictors or removing existing ones.

Regarding the computing performance of APRANK, the majority of the time is dedicated to run the predictors used internally, most of which run in a reasonable time in a commodity server. However, there are a few bottlenecks (most notably predictions by NetSurfP). This should be improved in a future version in order to offer APRANK e.g. as a web-service. Future work will also explore the possibility to extend APRANK to also use data from other experimental (non-computable) sources, such as evidence of expression derived from proteomic or transcriptomic experiments.

Finally, APRANK is currently focused on finding linear epitopes, and likely missing most of the conformational ones. This is evidently a limitation, but also reflects the current imbalance on experimental validation of linear vs conformational epitopes. There is much more information on linear epitopes and hence the field is ripe to develop applications like APRANK. This also affected the selection of predictors, many of which are also biased to predict/analyze linear features, and the selection of validated antigen and peptide data, which were obtained mostly from peptide microarray data. Introduction of new predictors may increase the amount of conformational information used to rank epitopes, but finding and reporting conformational epitopes would entail large changes to how APRANK currently works. While we believe that the best path forward for APRANK is to focus future work in increasing the accuracy of the prediction for linear epitopes, we do not rule out the possibility of adding the detection of conformational epitopes to this method.

### Equations

See **Supplementary Materials**.

## Data Availability Statement

The original contributions presented in the study are included in the article (see *Availability* in *Methods*) and the **Supplementary Material**. Further inquiries can be directed to the corresponding author.

## Author Contributions

Conceptualization: AR, SC, and FA. Data curation: AR, MB, and DR. Formal analysis: AR, MN, and FA. Funding acquisition: FA. Investigation: AR. Methodology: AR, MB, DR, and MN. Project administration: FA. Resources: FA. Software: AR, MB, and DR. Supervision: SC and FA. Validation: AR. Visualization: AR. Writing – original draft: AR and FA. Writing – review & editing: AR, MN, and FA. All authors contributed to the article and approved the submitted version.

## Funding

Research reported in this publication was supported by the National Institute of Allergy and Infectious Diseases of the National Institutes of Health under award number R01AI123070 and by Agencia Nacional de Promoción Científica (Argentina) under award numbers PICT-2013-1193 and PICT-2017-0175.

## Disclaimer

The content is solely the responsibility of the authors and does not necessarily represent the official views of the National Institutes of Health.

## Conflict of Interest

The authors declare that the research was conducted in the absence of any commercial or financial relationships that could be construed as a potential conflict of interest.
