# Supplementary material for: APRANK: Computational Prioritization of Antigenic Proteins and Peptides From Complete Pathogen Proteomes
Source: Front Immunol. 2021 Jul 15;12:702552. doi: 10.3389/fimmu.2021.702552 (PMC8320365; doi:10.3389/fimmu.2021.702552)
Supplement: Supplementary file 1 [file DataSheet_1.zip › ricci-et-al-aprank-supplementary-figures-tables-formulas.pdf]

# *Supplementary Material*

## 1 SUPPLEMENTARY DATA

### 1.1 Validated antigenic proteins and peptides

Antigenic Proteins and Peptides used in this study in training and validation of APRANK were submitted separately as Supplementary Material accompanying this article. The corresponding file is an Excel spreadsheet containing complete listing of antigenic sources (proteins, peptides), their Uniprot and/or RefSeq identifiers and the corresponding mapping to our input sources (complete proteomes).
File: ricci-aprank-validated-antigens-supplementary-data.xlsx. (Size: 302 KB)

### 1.2 Antigenicity scores for proteins and peptides

The antigenicity scores returned by APRANK for Proteins and Peptides were submitted separately as Supplementary Data to Dryad (DOI:10.5061/dryad.zcrjdfnb1, see Supplemental Information). The corresponding file is a compressed Zip file containing APRANK predictions for each organism. A README file explains the models used to calculate APRANK's scores in each scenario.
File: ricci-aprank-antigenicity-scores-supplementary-data.zip (Size: 59.3 MB)

## 2 SUPPLEMENTARY TABLES AND FIGURES

### 2.1 Figures

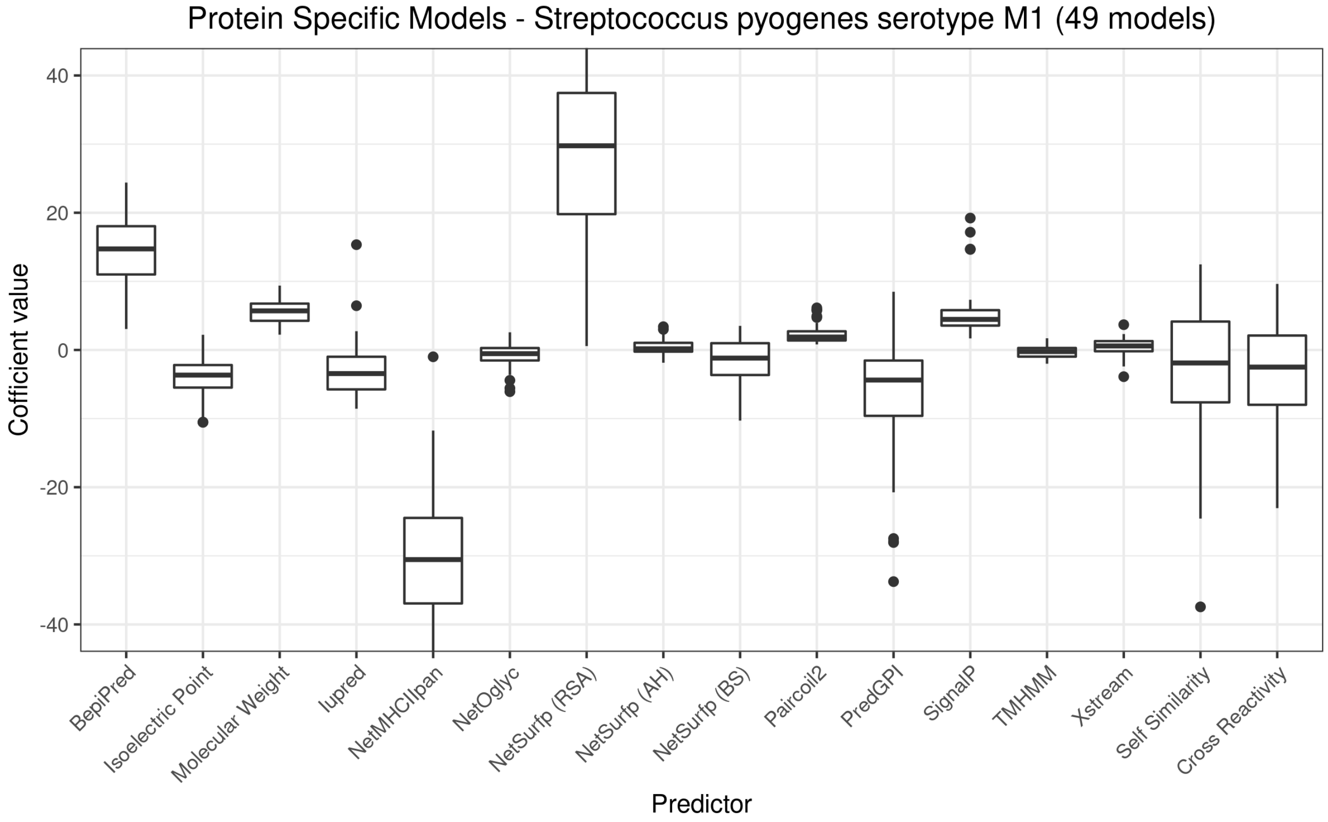


**Figure S1. Coefficient values for the species-specific models for *Streptococcus pyogenes serotype M1*.** Plots were obtained by recording the coefficient of each predictor in the binomial logistic regression models. These protein models correspond to the different species-specific models created when re-sampling training and test sets. One of the 50 models didn't converge before reaching the maximum iteration limit when training, and so wasn't considered.

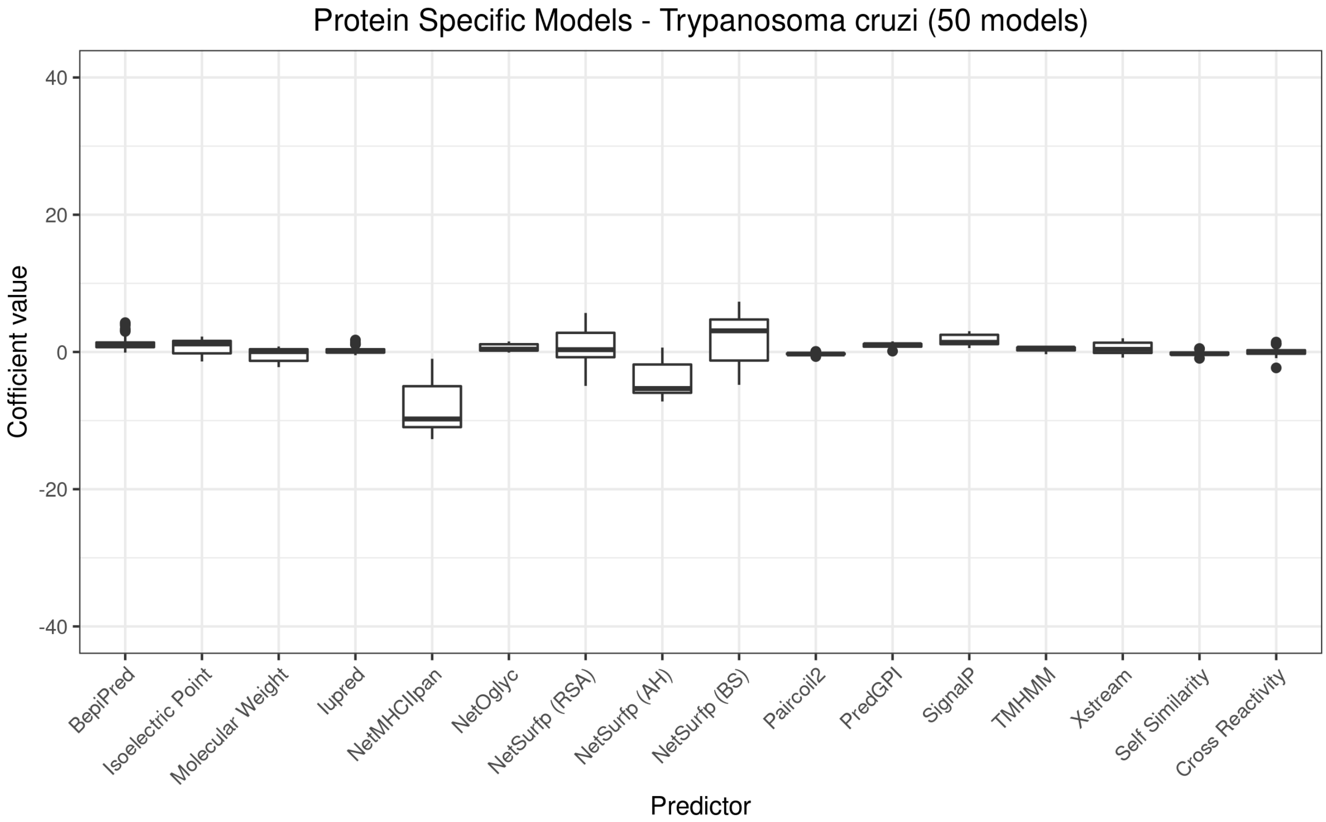


**Figure S2. Coefficient values for the species-specific models for *Trypanosoma cruzi*.** Plots were obtained by recording the coefficient of each predictor in the binomial logistic regression models. Different protein models correspond to the species-specific models created in each iteration when re-sampling training and test sets. All 50 models converged before reaching the maximum iteration limit when training.

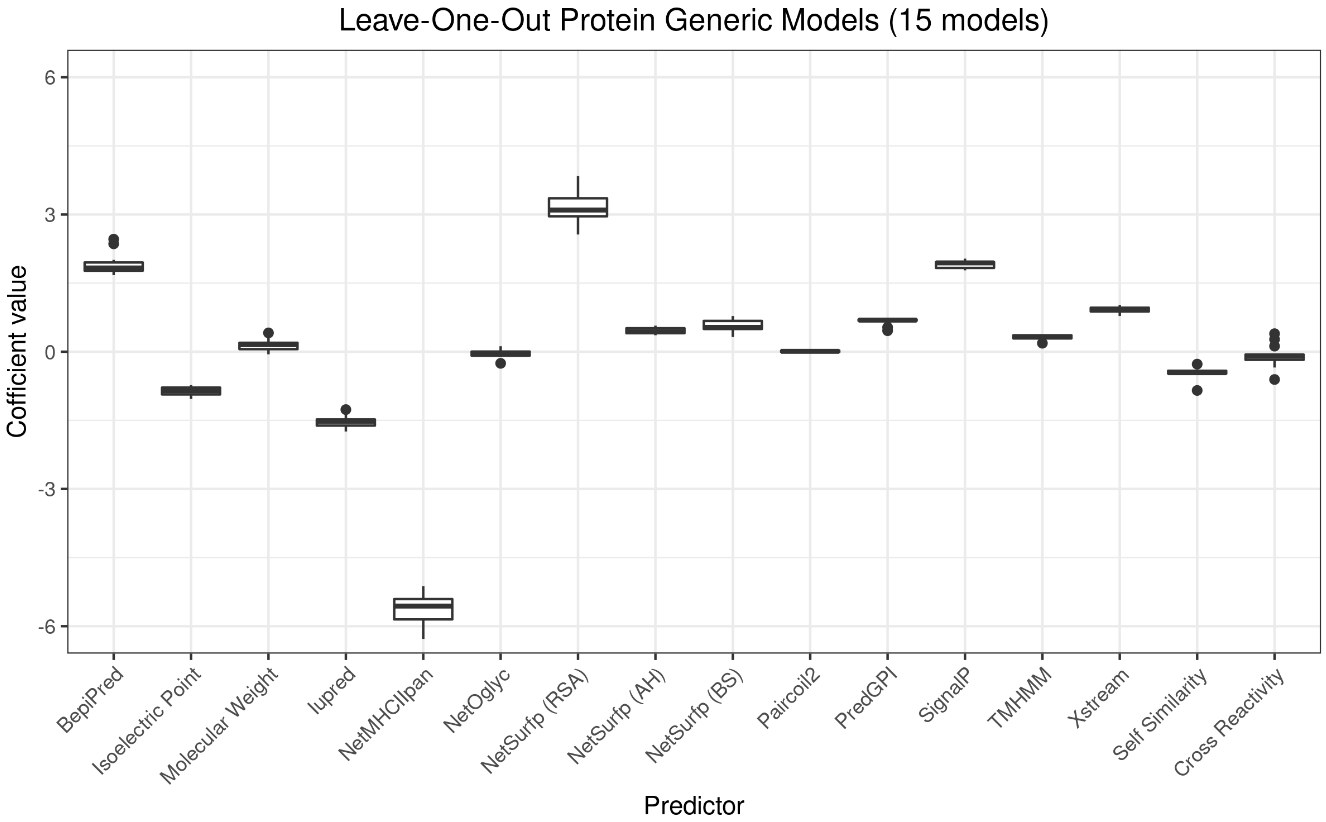


**Figure S3. Coefficient values for the leave-one-out generic models**. Plots were obtained by recording the coefficient of each predictor in the binomial logistic regression models. The different protein models correspond to each of the 15 leave-out-out generic models used to test APRANK. All 15 models converged before reaching the maximum iteration limit when training.

## 2.2 Tables

**Table S1. Versions testing things of the software, packages and modules used to create our computational method.**

| Software | Version |
|---|---|
| Ubuntu | 16.04 |
| R | 3.4.3 |
| ROSE (R package) | 0.0.3 |
| pROC (R package) | 1.12.1 |
| Perl | 5.22.1 |
| BioPerl (Perl module) | 1.007002 |

**Table S2. Third-party software used to retrieve information about the proteins and peptides.** The call being shown corresponds to those to use under Ubuntu 16.04. Words starting with $ symbolize variables to be replaced by their corresponding values.

| Predictor | Call | Data extracted |
|---|---|---|
| BepiPred 1.0 | bepipred $fasta_file -k >$output_file | Score per amino acid |
| BLAST+ 2.2.31 | blastp -query $query_file -db $db_file -outfmt 6 -out $output_file -max_target_seqs 2000 | Similarity between proteins (used to assign protein antigenicity) |
| EMBOSS 6.6.0.0 | pepstats -sequence $sequence_file -sprotein1 -aadata Eamino.dat -mwdata Emolwt.dat -termini -nomono -auto -outfile $output_file | Isoelectric Point and Molecular Weight per protein |
| Iupred 1.0 | iupred $sequence_file short >$output_file | Score per amino acid |
| NetMHCIIpan 2.0 | netMHCIIpan -a $allele -f $sequence_file -l $peptide_length >$output_file | %Rank per peptide per MHC II allele used |
| NetOglyc 3.1d | netOglyc $sequence_file >$output_file | Glycosilation presence per amino acid |
| NetSurfp 1.0 | NetSurfp $sequence_file -a >$output_file | Relative Surface Accessibility, Probability for Alpha-Helix and Probability for Beta-strand per amino acid |
| Paircoil2 | paircoil2 $fasta_file $output_file $error_file | P-score per amino acid |
| PredGPI 1.4.3 | PredGPI.py $filtered_fasta_file >$output_file | Presence and start of GPI per protein |
| SignalP 4.0 | signalp -f long -t $organism_group $fasta_file $output_file | Presence and start of signal peptide per protein and C and S score per amino acid |
| TMHMM 2.0c | tmhmm $fasta_file >$output_file | Presence of transmembrane helix per protein and amino acid participation in it per amino acid |
| Xstream 1.71 | java -jar $Xstream_path/xstream.jar $sequence_file -d$output_path/ | Start, end, period, copy number and consensus error per repeat per protein |

**Table S3. Normalization methods used for each predictor in protein and peptide analysis.** The formulas mentioned are shown in the supplementary materials.

| Predictor's output | Protein | Peptide |
|---|---|---|
| BepiPred | Calculate the mean of the BepiPred score for the amino acids inside the protein and normalize it using fixedLinearNormalization with -1.5 and 1.5 as limits | Calculate the mean of the BepiPred score for the amino acids inside the peptide and normalize it using fixedLinearNormalization with -1.5 and 1.5 as limits |
| Isoelectric Point | Divide the isoelectric point value by 14 | - |
| Molecular Weight | Normalize the molecular weight value using sigmoidNormalization05 with a b of 30,000. | - |
| Iupred | Calculate the ratio of amino acids inside the protein with an score greater or equal than 0.5 | Calculate the ratio of amino acids inside the peptide with an score greater or equal than 0.5 |
| NetMHCIIpan | Calculate the mean of the ranks for the kmers inside the protein, divide it by 100, normalize it using fixedLinearNormalization with 0.05 and 0.5 as limits, and then substract that number from 1 | Calculate the mean of the ranks for the kmers inside the peptide, divide it by 100, normalize it using fixedLinearNormalization with 0.05 and 0.5 as limits, and then substract that number from 1 |
| NetOglyc | Calculate the ratio of glycosilated amino acids inside the protein, and normalize it using fixedLinearNormalization with 0 and 0.05 as limits | Check if the peptide has at least 1 glycosilated residue |
| NetSurfp (RSA) | Calculate the mean of the values for the amino acids inside the protein | Calculate the mean of the values for the amino acids inside the peptide |
| NetSurfp (Alpha Helix) | Calculate the mean of the values for the amino acids inside the protein | Calculate the mean of the values for the amino acids inside the peptide |
| NetSurfp (Beta Strand) | Calculate the mean of the values for the amino acids inside the protein | Calculate the mean of the values for the amino acids inside the peptide |
| Paircoil2 | Check if the protein has an amino acid sequence of a given length (50 by default) where all the amino acids has a score above a threshold (0.5 by default) | Calculate the ratio of amino acids inside the peptide with a score above a threshold (0.5 by default) |
| PredGPI | Check if the protein has a GPI | Check if the peptide is at least in part inside the GPI |
| SignalP | Check if the protein has a signal peptide | Check if the peptide is at least in part inside the signal peptide |
| TMHMM | Use the output as it is | Check if the peptide is at least in part inside a transmembrane helix |
| Xstream | Find the largest copy number in the protein and normalize it using sigmoidNormalization09 with a b of 5 | Assign to each amino acid the highest copy number it's involved in, calculate the mean of that value for the amino acids inside the peptide and normalize it using sigmoidNormalization09 with a b of 5 |
| Cross Reactivity | For each kmer in the protein, normalize the amount of times that kmer appears in the host proteome using sigmoidNormalization05 with a b of 1, then calculate the mean of these values | For each kmer in the peptide, normalize the amount of times that kmer appears in the host proteome using sigmoidNormalization05 with a b of 1, then, calculate the mean of these values |
| Self Similarity | For each kmer in the protein, normalize the amount of other times that kmer appears in the proteome using sigmoidNormalization05 with a b of 1, then calculate the mean of these values | For each kmer in the peptide, normalize the amount of other times that kmer appears in the proteome using sigmoidNormalization05 with a b of 1, then, calculate the mean of these values |

**Table S4. Antigenicity discerning capabilities of predictors.** The table shows the net number of species where the mean of the normalized outputs for a given predictor are significantly different for the validated antigens than for the whole proteome (Student's t-test, $p < 0.05$). For each organism, if the mean was significantly greater for the antigenic proteins than for the whole proteome then we added 1, and if the mean was significantly less then we subtracted 1 (this is shown inside the parenthesis). The numbers in the columns correspond to the total amount of analyzed species inside that group.

| **Predictor** | **Antigenicity discerning capability** | | | |
|---|---|---|---|---|
| | **All (15)** | **Gram - (7)** | **Gram + (4)** | **Eukaryote (4)** |
| BepiPred | 12 (+12 -0) | 5 (+5 -0) | 4 (+4 -0) | 3 (+3 -0) |
| Isoelectric Point | -7 (+0 -7) | -2 (+0 -2) | -2 (+0 -2) | -3 (+0 -3) |
| Molecular Weight | 0 (+4 -4) | 0 (+1 -1) | 2 (+2 -0) | -2 (+1 -3) |
| Iupred | 4 (+5 -1) | 1 (+1 -0) | 2 (+2 -0) | 1 (+2 -1) |
| NetMHCIIpan | -13 (+0 -13) | -5 (+0 -5) | -4 (+0 -4) | -4 (+0 -4) |
| NetOglyc | 3 (+4 -1) | 2 (+2 -0) | -1 (+0 -1) | 2 (+2 -0) |
| NetSurfp (RSA) | 9 (+9 -0) | 5 (+5 -0) | 2 (+2 -0) | 2 (+2 -0) |
| NetSurfp (Alpha Helix) | -1 (+2 -3) | 0 (+1 -1) | 0 (+0 -0) | -1 (+1 -2) |
| NetSurfp (Beta Strand) | 2 (+3 -1) | 1 (+1 -0) | 0 (+0 -0) | 1 (+2 -1) |
| Paircoil2 | 2 (+2 -0) | 0 (+0 -0) | 1 (+1 -0) | 1 (+1 -0) |
| PredGPI | -5 (+2 -7) | -4 (+0 -4) | -3 (+0 -3) | 2 (+2 -0) |
| SignalP | 10 (+10 -0) | 5 (+5 -0) | 3 (+3 -0) | 2 (+2 -0) |
| TMHMM | 1 (+3 -2) | 1 (+2 -1) | 0 (+0 -0) | 0 (+1 -1) |
| Xstream | 5 (+6 -1) | 0 (+1 -1) | 2 (+2 -0) | 3 (+3 -0) |
| Cross Reactivity | 2 (+2 -0) | 1 (+1 -0) | 1 (+1 -0) | 0 (+0 -0) |
| Self Similarity | -3 (+0 -3) | -2 (+0 -2) | 0 (+0 -0) | -1 (+0 -1) |

**Table S5. Comparison between APRANK and a version of APRANK without the predictor with highest solo AUC (BepiPred 1.0).** The relative AUC gain shows the increase or decrease of the AUC obtained by APRANK relative to the version of APRANK without BepiPred. In bold we show differences greater than 5%. Due to the large number of peptides, each individual peptide AUC was calculated as the mean of 5 pseudo-random subsets of 50,000 peptides (see Methods).

| **Species** | **Group** | **Proteins APRANK score** | | | **Peptides APRANK score** | | |
|---|---|---|---|---|---|---|---|
| | | **without BepiPred AUC** | **with BepiPred AUC** | **Relative AUC gain** | **without BepiPred AUC** | **with BepiPred AUC** | **Relative AUC gain** |
| B. burgdorferi | Gram - | 0.777 | 0.786 | 1.18% | 0.726 | **0.768** | **5.78%** |
| B. melitensis | Gram - | 0.749 | 0.774 | 3.39% | - | - | - |
| C. burnetii | Gram - | 0.616 | 0.620 | 0.61% | - | - | - |
| E. coli | Gram - | 0.751 | 0.754 | 0.42% | 0.743 | 0.742 | -0.07% |
| F. tularensis | Gram - | 0.714 | 0.698 | -2.15% | - | - | - |
| L. interrogans | Gram - | 0.938 | 0.947 | 0.96% | 0.646 | **0.679** | **5.15%** |
| P. gingivalis | Gram - | 0.847 | 0.854 | 0.75% | 0.626 | **0.665** | **6.19%** |
| M. leprae | Gram + | 0.750 | 0.758 | 1.04% | 0.657 | **0.692** | **5.37%** |
| M. tuberculosis | Gram + | 0.697 | 0.702 | 0.66% | 0.586 | 0.586 | 0.00% |
| S. aureus | Gram + | 0.762 | 0.737 | -3.31% | 0.751 | 0.752 | 0.19% |
| S. pyogenes | Gram + | 0.983 | 0.983 | 0.04% | 0.826 | 0.838 | 1.47% |
| L. braziliensis | Eukaryote | 0.687 | 0.709 | 3.20% | 0.928 | 0.946 | 1.88% |
| P. falciparum | Eukaryote | 0.801 | 0.807 | 0.84% | 0.753 | 0.748 | -0.73% |
| T. gondii | Eukaryote | 0.835 | 0.837 | 0.27% | 0.585 | 0.583 | -0.47% |
| T. cruzi | Eukaryote | 0.869 | 0.867 | -0.29% | 0.833 | 0.843 | 1.26% |

**Table S6. Comparison between APRANK and a 'BLAST model'.** The 'BLAST model' worked by assigning to each protein a score related to how similar they were to a recorded antigenic protein. For the two species that resulted in a better prediction when using the 'BLAST model', we also tested removing from the BLAST results (and so, from the 'model') the species that was the most similar to the one being analyzed. In bold we show differences greater than 5%.

| **Species** | **Proteins** | | |
|---|---|---|---|
| | **BLAST AUC** | **APRANK AUC** | **Relative AUC gain** |
| B. burgdorferi | 0.502 | 0.786 | **56.60%** |
| B. melitensis | 0.637 | 0.774 | **21.49%** |
| C. burnetii | 0.579 | 0.620 | **7.14%** |
| E. coli | 0.677 | 0.754 | **11.44%** |
| F. tularensis | 0.629 | 0.698 | **10.92%** |
| L. interrogans | 0.499 | 0.947 | **89.86%** |
| P. gingivalis | 0.544 | 0.854 | **57.04%** |
| M. leprae | 0.893 | 0.758 | **-15.10%** |
| M. tuberculosis | 0.591 | 0.702 | **18.78%** |
| S. aureus | 0.622 | 0.737 | **18.56%** |
| S. pyogenes | 0.542 | 0.983 | **81.26%** |
| L. braziliensis | 0.951 | 0.709 | **-25.42%** |
| P. falciparum | 0.594 | 0.807 | **35.77%** |
| T. gondii | 0.443 | 0.837 | **88.98%** |
| T. cruzi | 0.501 | 0.867 | **72.95%** |
| M. Leprae (without M. tuberculosis in BLAST) | 0.650 | - | **16.63%** |
| L. braziliensis (without T. cruzi in BLAST) | 0.744 | - | -4.74% |

## 3 FORMULAS

$$fixedLinearNormalization(x, m, M) = \begin{Bmatrix} 0 & for\ x \leq m \\ \frac{x-m}{M-m} & for\ m < x < M \\ 1 & for\ x \geq M \end{Bmatrix} \tag{S1}$$

$$sigmoidNormalization05(x, b) = -1 + \frac{2}{1 + 3^{-\frac{x}{b}}} \tag{S2}$$

$$sigmoidNormalization09(x, b) = -1 + \frac{2}{1 + 20^{-\frac{x}{b}}} \tag{S3}$$
